# Impact of invasive VNS on depression severity and the need of concomitant drug and neuromodulatory treatment dose

**DOI:** 10.1192/j.eurpsy.2022.264

**Published:** 2022-09-01

**Authors:** E. Kavakbasi, B. Baune

**Affiliations:** University Hospital Muenster, Department Of Psychiatry, Münster, Germany

**Keywords:** difficult-to-treat depression, vagus nerve stimulation, VNS, DTD

## Abstract

**Introduction:**

Invasive vagus nerve stimulation (VNS) is an adjunctive long-term treatment option for chronic and recurrent difficult-to-treat depression (DTD).

**Objectives:**

In this prospective observational open-label case series we report on the effects of invasive VNS on depression severity, medication load and the need of maintenance electroconvulsive therapy (ECT) and esketamine treatment after 12 months.

**Methods:**

Patients were treated with invasive VNS according to clinical indication. All patients were included in the Restore-Life-Study. The assessment of depression severity (MADRS) and concomitant treatment was performed at baseline and in 3-months intervals postoperatively over a 12 months period.

**Results:**

Twelve patients were treated with adjunctive VNS due to unipolar (n=10) and bipolar (n=2) depression. The majority of patients were female (n=9). The mean age at baseline was 53.8 years (range 38-66). Patients were severely affected by a variety of depression symptoms which was reflected in high MADRS Scores (median 29, mean 28) at baseline. All patients received at least 2 or more psychotropic drugs at baseline. After 12 months of VNS a clear reduction of MADRS Scores (41 % on average) was seen (12-months MADRS Score median 17, mean 18). After 12 months, one patient each was discontinued from maintenance ECT and esketamine, respectively. The median of drug load was reduced from 4,56 to 4,06 after 12 months.

**Conclusions:**

Invasive VNS is an effective treatment option in the long-term management of DTD to reduce the need of concomitant drug dose and maintenance treatment.

**Disclosure:**

E Kavakbasi received speaker fees from Livanova. BT Baune received speaker and advisor fees from Livanova. The patients were included in the Restore-Life Study sponsored by LivaNova.

